# Understanding consumer attitudes towards second-hand robots for the home

**DOI:** 10.3389/frobt.2024.1324519

**Published:** 2024-07-10

**Authors:** Helen McGloin, Matthew Studley, Richard Mawle, Alan Frank Thomas Winfield

**Affiliations:** 1 FARSCOPE Centre for Doctoral Training, University of Bristol and University of West England, Bristol, United Kingdom; 2 School of Engineering, College of Arts, Technology and Environment, University of West England, Bristol, United Kingdom; 3 School of Architecture and Environment, College of Arts, Technology and Environment, University of West England, Bristol, United Kingdom

**Keywords:** robotics, sustainability, circular-economy, second-hand, e-waste, repurpose, reuse, recycle

## Abstract

As robot numbers in the home increase, creating a market for second-hand robotic systems is essential to reduce the waste impact of the industry. Via a survey, consumer attitudes of United Kingdom participants towards second-hand robots were investigated; finding that second-hand robots with guarantees have an equal purchasing interest compared to new systems, highlighting the opportunity for manufacturers and retailers to develop certification standards for second-hand robots to move towards a circular economy. Consumer demographics also demonstrated that those most open to the purchase of both new and second-hand systems were women, those aged 18–25 years old, and those who have previously owned a robot for the home. Participants’ prior ownership of second-hand electronic devices (such as phones and laptops) did not affect rates of interest for second-hand robotic systems suggesting that the technology is still too new for people to be able to project their experience of current second-hand electronics to that of a robot. Additionally, this research found the robotics industry can consider the potential market for second-hand robots to be more similar to the second-hand smartphone market than to the household electronics market, and lessons learnt from the concerns raised by consumers for other internet-enabled electronic devices are similar to those concerns for second-hand robots. This provides an opportunity for the industry to break down the barriers for a circular economy earlier in the technology maturity process than has been seen for other electronics.

## 1 Introduction

First published in 2015, the United Nations announced 17 Sustainable Development Goals [UN SDG] as part of their 2030 agenda for sustainable development providing a “plan of action for people, planet and prosperity” United Nations (2015). SDG 12 aims to “ensure sustainable production and consumption patterns” to tackle the impacts of open loop consumption United Nations (2015). These impacts include climate change, biodiversity loss and deforestation, and pollution which harms people, animals and habitats United Nations (2015); Dauvergne and Lister (2010); Carlisle and Hanlon (2007). With 80 percent of a product’s environmental impact decided during the design and development phases of a product’s life cycle Charter (2019) and human open-loop consumption impacting so heavily on climate change Łukasz et al. (2022), building sustainable consumption patterns requires the buy-in and leadership of the product manufacturers to develop systems which are better suited to closed-loop consumption, also referred to as the ‘Circular Economy’ UKRI (2021); Elzinga et al. (2020).

Many electronic product manufacturers rely on the concept of recycling as a method to tackle open-loop consumption. However, across the globe, there is evidence of huge inefficiencies in the process of recycling. Globally, in 2019, only 17.4 percent of electronic waste created annually was recycled through formally managed systems Forti et al. (2020). Individuals living within the EU produce the highest levels of e-waste globally at 16.2 kg *per capita* per year Forti et al. (2020). Recycling rates are also highest in the EU, though rates only reach 42.5 percent Forti et al. (2020). In comparison, annually (in 2019), individuals in Oceania produce 16.1 kg *per capita* and recycled 8.8 percent, in the Americas they produced 13.3 kg *per capita* and recycled 9.4 percent, Asia produced 5.6 kg *per capita* and formally recycled 11.7 percent, while in Africa *per capita*, e-waste production annually was 2.5 kg and recycling rates were 0.9 percent Forti et al. (2020).

Consumer robots, generally being systems which require electrical current or electromagnetic fields to meet their functional purpose, could be considered as Electronic or Electrical Equipment [EEE] under the current standard accepted definition 200 (EUR -Lex, 2003). Therefore, when a consumer robot reaches the end of its useful life it will also then be classed as Waste Electronic or Electrical Equipment [WEEE], also known as e-waste. And, taking the global data for recycling rates of other electronic products, it would be reasonable to assume that, without specific intervention from the robotics industry, recycling rates for robotic systems will be similarly low to those seen for other types of e-waste due to the consistently poor actions and habits of the general population in managing e-waste from the home.

Alternatives to recycling and landfill for robotic systems are; repurposing, reusing, remanufacturing, reconditioning and repairing the systems at the end of their primary life. These options extend beyond the traditional 3 R’s: Reduce, Reuse, Recycle HM Government (2018), recognising the additional alternatives of Repair, Reconditioning, Remanufacturing and Repurposing. Reuse, recondition and repair are well-established processes; remanufacturing is a less common but well-documented method outside of the robotics industry for breaking down a system to component level and rebuilding it as new Nasr (2019); Steinhilper (1998); Steinhilper et al. (2017); while repurposing is a new concept for robotic systems which is under investigation by the authors of this paper McGloin et al. (2023).

Each of these alternative methods to recycling aims to increase the working life of a product, which in turn reduces waste production by delaying the time until the system needs to be recycled or sent to landfill, and forms the basis of a circular economy (UN SDG #12). In addition to this, the forming of a circular economy for robotic products for domestic settings will also; increase accessibility to technology through opportunities for lower-cost second-hand systems (UN SDG #8); reduce deforestation of land used for mining materials needed for production (UN SDG #15) and reduce emissions associated with the production of electronic products such as robots (UN SDG #13).

However, a critical element in making a circular economy viable lies in the participation of consumers in this business model and the ability to sell the resultant second-hand product to customers Elzinga et al. (2020). Consumers often require both push and pull influence factors to be in place to overcome established habits and be persuaded to purchase second-hand over new products Hazen et al. (2017). Push factors include the opportunity to purchase second-hand products at favourable rates to new ones, and the introduction of laws driving consumer habit changes. Pull factors might include tax breaks, government- or business-led incentives, and the purchaser’s personal perspective on environmental issues Hazen et al. (2017); Łukasz et al. (2022). Additionally, social media may create both push and pull influence factors for consumer purchasing habits. The aim of this study, therefore, was to assess the difference in consumer attitudes towards the purchase of new and second-hand robotic systems for domestic settings and to identify key factors which could progress or hinder the uptake of second-hand robots amongst potential users of robots for the home. This research was carried out using a survey method described in Section 2.

Understanding potential consumer behaviours towards second-hand robotic systems provides an opportunity for researchers, developers and manufacturers of robots for the home to build more sustainable practices into the products they create before they become ubiquitous. Once in an accepted ubiquitous state, lock-in factors inhibit changes by both consumers and the Original Equipment Manufacturers from easily making more sustainable practice choices Aminoff and Sundqvist-Andberg (2021); Ellen Macarthur Foundation. (2023). Additionally, the results of this research are part of a wider program which is also investigating the attitudes of the robotics industry towards sustainability, and the reuse and repurposing of robotic systems. The results of the industrial attitudes research will be presented in a separate paper.

## 2 Methodology

### 2.1 Research aim

The aim of this research was to assess consumer interest in second-hand robots–such as those which have been reconditioned or repurposed, in comparison to new systems, and to identify key factors which could affect the uptake of second-hand systems.

In order to meet the aims of the study, an appropriate research philosophy was selected (Section 2.2) which influenced the development of the study design (Section 2.3). Data was then collected (Section 3) and analysed (Section 4), with conclusions being presented in Section 5.

### 2.2 Research methodology

**FIGURE 1:**
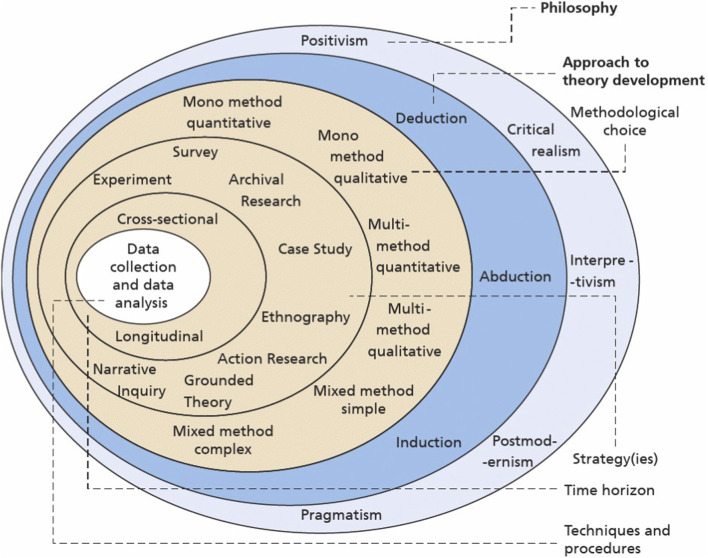
The ‘research onion’ (pg 130 Saunders et al. (2019). Source: Saunders MNK; Saunders et al. (2019) Research methods for Business Students (8th edition) Harlow: Pearson: p 130. The research onion is ⓒ 2018 Mark Saunders, Philip Lewis and Adrian Thornhill and is reproduced in this article with their written permission.

Saunders et al. (2019) present the ‘research onion’ (Figure 1) which summarises the methodological choices, strategies, data collection and analysis methods, which together form the widely accepted methodologies used in research strategies. The methodologies utilised in this research were;

An *Inductive* research approach due to there being limited existing data available from the robotics field that could produce a theory which might, in turn, validate or invalidate a hypothesis based on the data collected.

A *survey* method for data collection was selected as very few people already own robots, so observational data could not be collected for the purpose of this research. Instead, data collection relied on participants’ opinions based on the information provided to them and utilised a Likert Scale system with free-text open-response questions for additional participant responses. Likert Scales were selected because they are easy for participants to use, resulting in increased response rates and reliability Jupp (2006).

*Quantitative* analysis methods were selected for the Likert Scale survey data which was then transcribed from ordinal data to interval data during the analysis process. This transcription allows for the conversion of the opinions of participants into numerical-based data Fleetwood (2014).

*Qualitative* analysis methods were selected for the analysis of the free-text survey responses via Thematic analysis which aims to understand the core themes presented both in a participant’s individual responses and also between participants’ responses Bryman (2016).

### 2.3 Study design

Following the selection of the research methodology (Section 2.2), a survey was developed which was designed for access by the general public via the Qualtrics online platform. The survey was divided into the following sections:.

**FIGURE 2:**
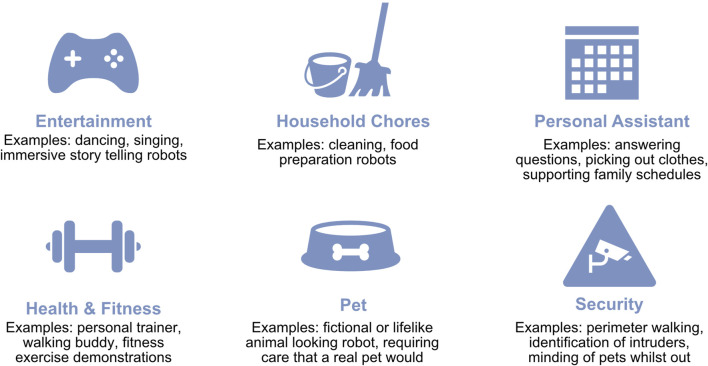
Types of consumer robots presented to participants in the online survey, along with examples of each robot type. Note, graphic not used in survey, only wording.

- **Demographics and lifestyle factors**–individuals were required to provide demographic data such as age range and country of residence, alongside a broad range of personal lifestyle indicator factors that the research team felt could influence the likelihood of uptake second-hand robotics. These lifestyle factors included attitudes to environmental topics, home ownership status, prior ownership of robotic systems in the home, uptake of internet-enabled technology devices in the home and previous purchasing decisions for home technology. In this survey, internet-enabled devices were defined as a device which requires connection to WiFi or mobile data in order to function, but does not include mobile phones, laptops, computers or tablets. All questions in this section provided participants with multiple-choice options for their responses. A concentration test question was also placed at the end of this section, to reduce the effect of random selection responses.
- **Purchasing attitudes**–participants were presented with a variety of robot types that could possibly be purchased in the future via different purchasing conditions; new, second-hand with a guarantee, and second-hand without a guarantee. For each robot type, a short description was given, and examples were also provided (see Figure 2). Participants were then asked for their opinions on if they would or would not be inclined to purchase the robots in the different purchase conditions. The proposed robot types included; robots used to provide security, perform household chores, to have as pets, work as personal assistants, and act as health and fitness instructors. Participants were asked to respond to questions using a three-point Likert Scale to demonstrate their interest in a given system.
- **Concern factors**–for each purchase condition, (new, second-hand with a guarantee and second-hand without a guarantee) participants were asked to rate their concern levels on a five-point Likert scale for factors including cost, security, environmental impact and safety. The five -point Likert Scale gave participants the option to respond Very Concerned, Slightly Concerned, Neutral, Slightly Unconcerned and Unconcerned for a given factor. In addition to this, a free-text answer box was provided to collect additional comments from participants.

Lastly, the participant survey design included the presentation of a downloadable participant information sheet and a data privacy policy.

### 2.4 Data collection and analysis procedure

The participant study gained ethical approval through the University of West England. Following this, the study was shared through both social media platforms and via surveycircle–a website for finding survey participants. The survey was open to any individual who was over the age of 18 years old and consented to take part in the research. The responses submitted by participants were checked and rendered anonymous after 7 days.

Excel was used to analyse the demographic and Likert scale data, while NViVo was utilised for the analysis of free-text responses. The Nvivo analysis followed a Thematic approach (as described in Section 2.2). The method to complete the thematic analysis within Nvivo was:

- Phase I–Open Coding: this required the line-by-line analysis of raw data (from the survey free-text responses) to draw out concepts presented within the data Khaksar et al. (2015); Charmaz (2006). The purpose of this phase was to find meaning and actions behind the words given by the participant. Each concept was labelled (referred to as a code), to enable repeating concepts to be highlighted under the same code Hutchison et al. (2010). At this stage it was expected that as wide a range of coded concepts be identified as possible since fitting answers under pre-existing data labels would limit the analysis and stop new ideas from emerging Charmaz (2006)). The codes were considered provisional and could be amended at any time. The overall aim was to “make the codes fit the data” rather than “forcing the data to fit” the codes Charmaz (2006).
- Phase II–Thematic framework: during this phase, codes were grouped into categories and sub-categories and the links between the codes which form categories were noted Khaksar et al. (2015). In this way, the data which was analysed at a line-for-line level in the open coding was brought back together and reassembled by identifying the connections between the codes Hutchison et al. (2010), thereby highlighting the key themes in the research.

### 2.5 Participant demographics and lifestyle factors

A total of 111 individuals responded to the online survey, including responses from 16 individuals who did not fully complete the survey and whose responses were removed from further data analysis. Of the remaining responses, 72 were from individuals in the United Kingdom and 23 were from individuals outside of the United Kingdom. Those responses from individuals outside of the United Kingdom covered nine different countries and, for the purpose of the study presented within this paper, were not included in the data analysis set due to the small sample size.

Of the 72 responses from United Kingdom residents carried forward for analysis, 60 percent of participants were female, compared to 51 percent of the United Kingdom populationGov (Ethnicity, 2018). A comparison of the age demographic of the survey participants versus the United Kingdom population is shown in Table 1. The age group with the greatest over-representation in comparison to the United Kingdom population was those aged 26–35, while the greatest under-representation was in the 76+ age group which highlights the limitation of using online platforms to both advertise and complete the survey. Additionally, the participants for the study represented the following demographics:

- **Home ownership rate**–67 percent of participants lived in owned (with or without a mortgage) accommodation, versus 63 percent of people in the United Kingdom Gov Ministry of Housing (2020).
- **At-home dependants**–29 percent of participants lived in homes with children under the age of 18 in them, compared to 45 percent of people in the United Kingdom living in households containing one or more dependant child ONS (2022).
- **Climate emergency beliefs**–96 percent of participants who completed the survey selected that they believe there is a climate emergency, versus 71 percent in the general United Kingdom population Booth-Dale (2022).

**TABLE 1:** Percentage of population in each age group (listed in years).

Age group (years)	18–25 (%)	26–35 (%)	36–45 (%)	46–55 (%)	56–65 (%)	66–75 (%)	76+
Survey Participants, n = 72	13	29	21	11	18	7	1%
United Kingdom Population Statista (2021)	11	17	16	16	16	13	12%

## 3 Results

### 3.1 Purchasing habits by consumers for non-robotic products

To better understand the attitudes of participants towards second-hand robots, prior electronic purchasing habits were investigated as part of the participant demographics. Excluding mobile phones and laptops, participants were asked for the number of internet-enabled devices in their homes. The results of this are shown in Table 2.

**TABLE 2:** Numbers of internet-enabled devices in the homes of participants.

Number of internet-enabled devices	Zero (%)	1–3 (%)	4–6 (%)	7–9 (%)	10+
Percentage of participants (n = 72)	15	47	21	4	13%

The majority of participants (47 percent) owned one to three internet-enabled devices in their homes, while only 15 percent did not own any internet-enabled devices. Those without internet-enabled devices were generally women (23 percent of the women surveyed did not have internet-enabled devices in the home compared to 4 percent of men).

**FIGURE 3:**
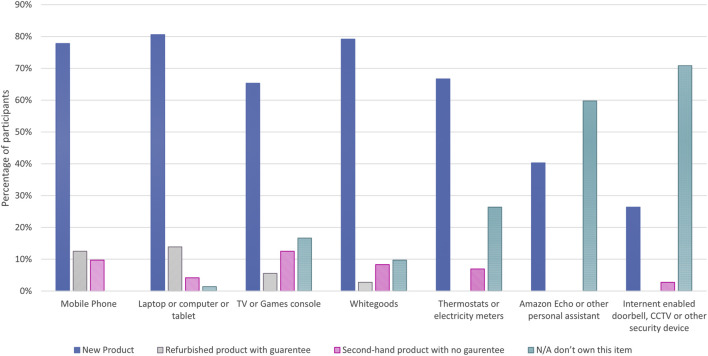
Conditions which participants bought other electronic items.

Participants’ willingness to purchase second-hand electronics was established through survey questions which required them to identify the condition in which they last bought a given type of electronic device (Figure 3). Purchase of electronics second-hand with guarantees was highest for the items in which there are multiple outlets available to make that type of purchase, such as mobile phones and laptops. Overall, second-hand purchases (with or without a guarantee) accounted for 23 percent of mobile phone purchases, 22 percent of TV or games console purchases, 18 percent of laptop or computer or tablet purchases, 10 percent of internet-enabled security devices and 9 percent of thermostat purchases. The only category where no participants had bought second-hand devices was the smart assistant devices (such as Amazon Echo or Google Home). Ownership levels for the different electronic devices varied, with all participants owning a mobile phone and only a single participant not owning a laptop, computer or tablet. 17 percent of respondents indicated they had not purchased a TV or games console, with 27, 59 and 70 percent of participants respectively indicating they had not purchased electronic thermostats, smart assistance devices and internet-enabled security devices. It should be noted though that TV ownership in the United Kingdom shows only 3 percent of households in the United Kingdom do not have a TV Statista (2022) compared to the 17 percent of survey participants. However, Statista (2022) statistics do highlight that only 70 percent of United Kingdom households own a smart TV. It is possible that either the participants had lower TV ownership than in comparison to the United Kingdom, or, more likely, that the writing of the survey did not highlight that the TV did not need to be internet-enabled and this requirement was assumed by participants due to the prior question on internet-enabled devices.

### 3.2 Consumer attitudes towards purchasing of second-hand robots

**FIGURE 4:**
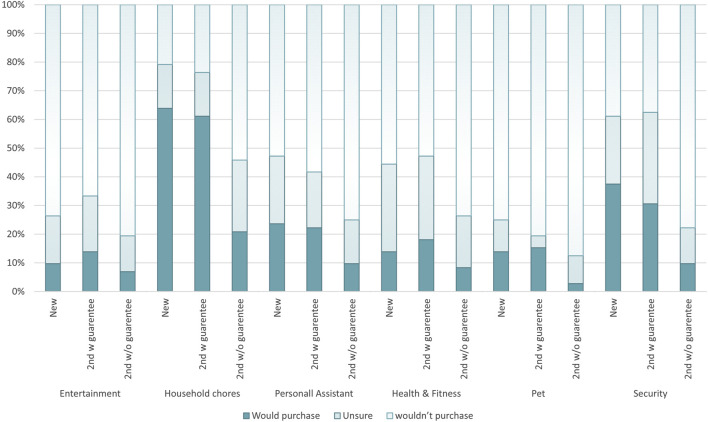
Purchase indication by participants for a given robot type, based on the purchase conditions: New, Second-hand with guarantee [2nd w guarantee], Second-hand without guarantee [2nd w/o guarantee].

Across all robot types presented to participants, 27 percent of participants indicated they would purchase a robot new, 27 percent would purchase one second-hand with a guarantee but only 10 percent would purchase one without a guarantee. The specific type of consumer robot presented to the participants affected the indicated purchase rate, with the highest interest seen for household chores robots at 64 percent for a new system, and the least interest was shown for a second-hand pet robot without a guarantee at 3 percent of participants indicating they would be willing to purchase such a system. In Figure 4 the purchase indication rates for each robot type and condition are presented.

**FIGURE 5:**
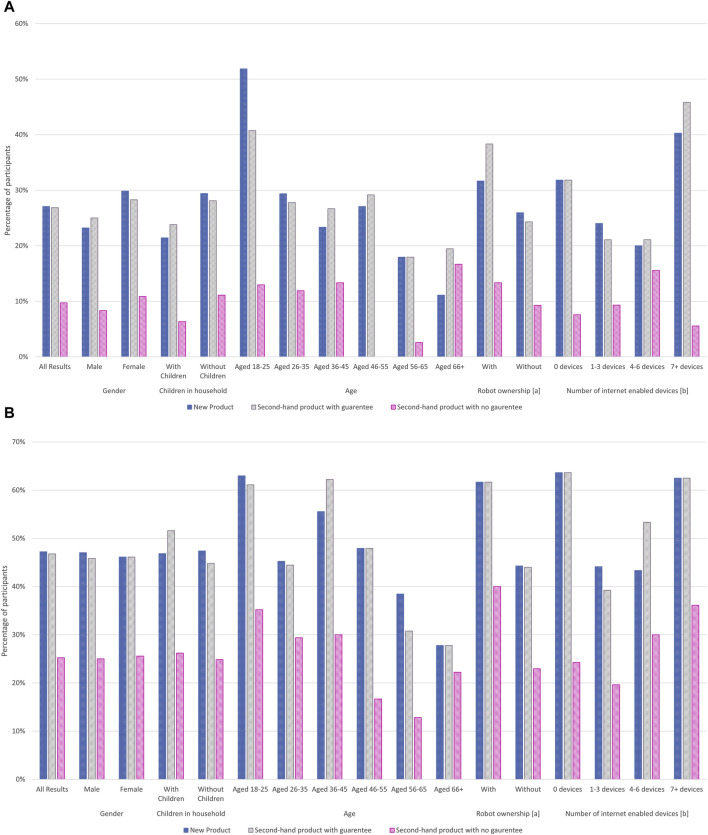
Effect of demographics on participants responding if they would purchase a robot based on the purchase conditions: New, Second-hand with guarantee, Second-hand without guarantee. Notes: [a] Prior ownership includes vacuum or mower-type robotic systems for the home. [b] Numbers of internet-enabled devices includes connected devices already in the home]. **(A)** Participants responding positively to purchase a robot in a given condition. **(B)** Combined responses for participants responding positively and unsure to purchase a robot in a given condition.

For each demographic and lifestyle factor surveyed, the percentage of participants in each group indicating they would purchase a robot for a given sale condition is shown in Figure 5A, and is detailed in the Supplementary Material along with numbers for participants responding they were ‘unsure’ if they would purchase a robot of a given category.

Women were more likely than men to indicate a positive response towards purchasing a robot, regardless of the condition, and those without children were also more likely to purchase a robot in all condition types. Interest in the purchase of new systems new generally decreased with age, with a 52 percent interest for those aged 18–25 years, down to 11 percent for those aged 66+. Only those aged 36–45 did not follow this trend, with their interest levels being 4 percent lower than for 56–65 year olds. Individuals were also more likely to select that they would purchase a new or second-hand robotic system if they already owned a robot in the home (such as a vacuum cleaner or lawn mower) compared to those who did not.

Generally, a ± 3 percent difference was seen when comparing the attitudes of participants to new robots, to second-hand robotic systems with guarantees. Exceptions to this were for those aged 18–25 which had a drop of 11 percent between new and second-hand with guarantee, the age category 66+ years which saw an 8 percent increase, prior ownership of robots which had a 7 percent increase and those with seven or more internet-enabled devices in the home which had a 6 percent increase respectively.

Across all factors and purchase conditions investigated the most popular type of robot was a robot for household chores, generally followed by robots for security systems. Only in the second-hand with no guarantee condition were robotic security systems not the second-place preference option. Instead no trends were found shown in the responses across demographic factors for this condition, beyond the initial preference by participants for household chore robots.

Combining the positive and neutral responses (Figure 5B) resulted in response rates of over 40 percent for new robots in all demographic categories except those aged 56–65 and 66+. The same trend was seen for second-hand robots with guarantees but with the addition of those who have 1-3 internet-enabled devices in the home having a response rate of 39 percent.

### 3.3 Concern factors for consumers when purchasing second-hand robots

In addition to providing purchase indicators, concern factors recorded on a Likert scale were provided by participants. The response options were very concerned, slightly concerned, neutral, slightly unconcerned and unconcerned. Table 3 summarises the percentage of respondents selecting Very or Slightly concerned for each factor. The breakdown of the remaining Likert scale responses is given in the Supplementary Material.

**TABLE 3:** Percentage of participants selecting ‘Very concerned’ or ‘Slightly concerned’ for each factor described, based on a robot’s sale condition.

	Purchase new (%)	Second-hand with guarantee (%)	Second-hand no guarantee (%)
Cost of purchase	90	88	76
Cost to maintain	83	89	92
Physical safety for people in the home	56	67	69
Physical damage to the home	58	64	71
Data security for people in the home	85	90	86
Manufacturing environmental impact	72	63	65
Environmental impact of disposal	82	78	72

Those who own robots were generally less concerned by these factors than those who did not own robots. The greatest difference in responses of very or slightly concerned to new, second-hand with guarantee, and second-hand without guarantee was for the factors:

- Physical safety for people in the home (19, 32 and 35 percent difference respectively)
- Physical damage to the home (22, 17, 24 percent difference respectively)

The only instances where those who own robots had a greater level of concern shown than those who did not own robots were; the cost to maintain second-hand robots without guarantees (10 percent higher), the security of personal data and the environmental manufacturing impact on new robotic systems (6 and 8 percent higher).

Of the 72 participants in the study, 19 provided responses in the free-text sections of the survey requesting additional comments relating to concerns which had not been highlighted in the Likert Scale responses. Qualitative analysis of those free-text responses from participants identified over 32 additional areas of concern (codes), which together formed eight key themes:

- Concerns about the appearance of a robot
- Concerns around the purchasing source and guarantees for robots
- Concerns about the cost of a robot across its life-cycle
- Negative effects of robots on people during the robots use
- Concerns relating to the technical capability of robots
- Concerns around data security and privacy for robot owners
- People not wanting, or not able, to operate robots
- Impacts on society of greater robot use

**FIGURE 6:**
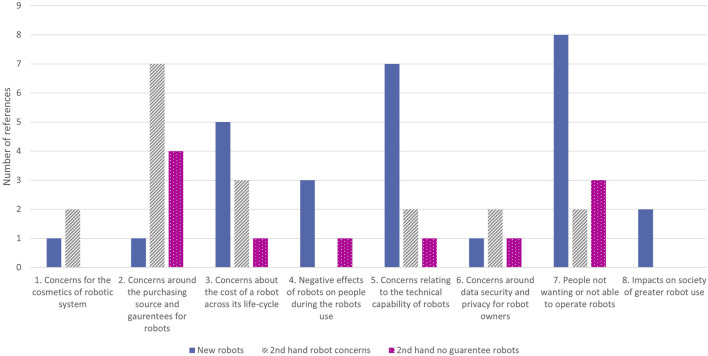
Graph of the number of coding references for each theme identified during the qualitative analysis.

Figure 6 summarises the number of references given for each of the themes identified, split by the condition of purchase. The theme with the greatest level of responses was those not wanting or not able to operate robots with comments from participants including; “I cannot imagine a circumstance where I would buy a new robot for my home.” [Participant #22], and concern about the purchase being “complicated operation or setting up of a unit” [Participant #25].

For new robots, the second greatest number of responses were related to the technical capability of the systems purchased. Comments within this theme included: “Could it cope with an old cottage with uneven floors” [Participant #18] and “How well would it actually do the job I purchased it to do?” [Participant #38].

For second-hand robots, both those with a guarantee and those without, respondents’ key concern centred on the purchasing source, continued support for the systems and guarantees relating to the second-hand product. Examples included; “problems with robot not identified or lied about” [Participant #59, comments for second-hand robots with no guarantee], “Spares and support. Mobiles are only supported for a few years. Given the likely cost of a robot I’d want 15 years or more, like cars.” [Participant #9, with guarantee], and “Credibility and viability of the organisation supporting product/guarantee” [Participant #56, with guarantee].

A variety of factors relating to cost were highlighted in the responses across all three robot conditions (new, second-hand and second-hand with guarantees), with concerns about ongoing maintenance, energy consumption and resale costs highlighted. Participants’ comments relating to the cost of systems included; “whether I would use it enough to justify buying it” [Participant #19, new robots], “I could be spending a lot of money and have no means of refund or exchange if the robot went wrong or ceased to work.” [Participant #38, second-hand no guarantee robots], “device energy consumption” [Participant #2–new] and “cost of software updates” [Participant #20–both new and second-hand with guarantee robots].

### 3.4 Comparison of purchasing indications for robots to other electronic products

**FIGURE 7:**
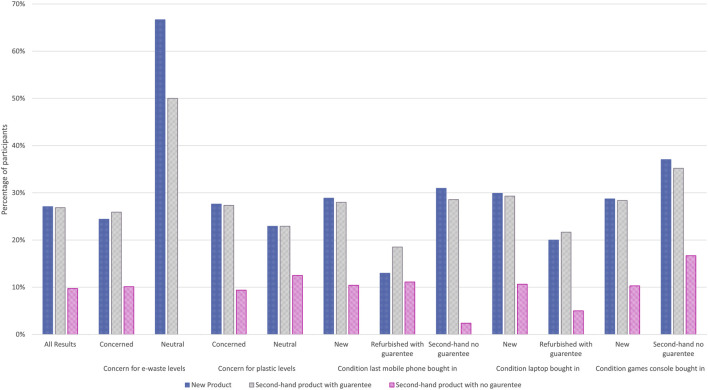
Effect of environmental lifestyle factors on participants responding if they would purchase a robot based on the purchase conditions: New, Second-hand with guarantee, Second-hand without guarantee.

Participant responses regarding concern levels for environmental issues were compared to robot purchasing conditions (shown in Figure 7). Those who indicated concern for e-waste levels were less likely to respond positively about purchasing new and second-hand robot products than those who indicated they were neutral towards concerns about e-waste. In comparison, those who were concerned about plastic waste levels were slightly more likely to respond positively about purchasing new and second-hand robot products than those who indicated they were neutral towards concerns about plastic waste levels. It was not possible to compare these results to responses about concern levels for the Climate Crisis, Deforestation and Pollution as the numbers responding neutral or unconcerned to these topics accounted for only 4 percent of the respondents (or three individual participants). Future surveys would need to address the recruitment and self-selection process found in this research that resulted in the majority of participants having high concern levels for topics such as the climate crisis, and therefore making sample sizes for those responding neutral or unconcerned too small to review.

Figure 7 also presents how the response by the participant on the last condition they purchased a mobile phone, laptop and games console affected their purchasing indications for robots in a given condition. Participants who had bought games consoles second-hand without a guarantee were more likely to positively respond to purchasing a robot in any condition type, while the opposite was true for those buying laptops new who had a higher robot purchase indication rate than for those who bought laptops refurbished with guarantees.

Supporting data for all figures included in Section 3 is included in the Supplementary Data S1.

## 4 Discussion

### 4.1 Consumer attitudes towards purchasing of second-hand robots

Consumer attitudes towards the purchase of second-hand robots with a guarantee matched interest levels for new systems at 27 percent, while only 10 percent of participants indicated a purchase possibility when there was no guarantee offered. Equal favourability for new and second-hand with guarantee robots does suggest that there is an opportunity for the sale of systems in both conditions. To enable the sale of second-hand systems though, vendors require the return of old systems at the end of their life requiring the industry to consider this in their business models. Elzinga et al. (2020) presents research into three business models for the electronics industry consumer circular economy; take-back management, product lease and pay-per-use, with take-back management the most popular option with consumers. The take-back management business model requires businesses to re-obtain ownership of products through collection points, with consumers incentivised to take part through payment schemes or fees Elzinga et al. (2020). This suggests that use of a take-back management scheme within the robotics industry will result in additional sales for robot manufacturers through the reconditioning and resale of second-hand systems, enabling a more circular economy.

The success of a take-back management scheme for the robotics industry would require easily accessible return points and well-communicated returns processes. These are particularly important to the electronics industry, where long-term storage of small household electronics regularly occurs and is a key barrier to electronics recovery Wilson et al. (2017). This process, also referred to as the hibernation phase of electronics ownership Murakami et al. (2010), is a well-documented consumer behaviour in developed countries. 70 percent of consumer e-waste was placed in hibernation for three to 5 years by consumers in the US Babu et al. (2007), while consumers in the United Kingdom stored mobile phones for an average of 3 years Wilson et al. (2017), and in a study of residents in a city in Finland–70 percent of participants retained their mobile phone for a period of time after they had no primary use for it in a study Ylä-Mella et al. (2015).

When considering the different types of robots presented to participants, interest was highest for robots which were able to complete household chores, followed by robots performing security roles. Overall interest levels amongst participants for new and second-hand with guarantee robots for chores were 62 percent and 58 percent respectively, while security robots had interest levels of 37 and 32 percent respectively. These results are comparable with the Nitto et al. (2017) study found that 50 percent of consumers in the US were interested in robots that would help with either household chores or security. This study did find though that levels of interest varied by country of residents, with 34 percent of residents of Germany and only 19 percent of residents from Japan indicating they would be interested in purchasing a robot within the next 5 years.

Robots used as pets were the least popular across all sale condition types, with a second-hand pet robot with no guarantee interesting only 3 percent of respondents. This was followed by robots for entertainment which had similar rates for new and second-hand with guarantee systems as pet robots, but a slightly higher interest in second-hand systems at 7 percent. Again, the Nitto et al. (2017) study noted that “when it comes to leisure, time spent with friends or caring for pets and children, American consumers are noticeably less interested in being involved with robots”, confirming the findings of this study.

Only robots used for entertainment, or for health and fitness, saw the rate of interest increase for a second-hand robot with a guarantee over a new robot, with the remaining categories seeing a decrease between the buying conditions. It is possible that due to the second-hand market for non-robotic technologies for both entertainment and fitness already being well established, there is a greater openness amongst participants to see second-hand robots in these sectors.

Demographics which most influenced a survey participants’ interest in purchasing new robots were age and prior ownership of robotic systems such as vacuums or mowers. 60 percent of those aged 18–25, and 37 percent of those previously owning a robotic system would purchase a robot new. Only those aged 66 and over were more likely to show an interest in purchasing a second-hand system (with or without a guarantee) rather than a new system. These results differed from those in the Pérez-Belis et al. (2017) study on consumer attitudes in Spain towards second-hand EEE purchases for the home which found that older consumers and women were more likely to repair small household EEE, while men and those from medium-income families were more likely to purchase second-hand small household EEE.

To better understand participants’ responses to second-hand robots, this survey also reviewed their prior purchasing habits of internet-enabled devices such as mobile phones, games consoles, personal assistant speakers and internet-enabled security doorbells or CCTV. For participants in this survey, 22 percent had owned a second-hand mobile phone and, of those who owned laptops, games consoles, and internet-enabled security systems, the percentage who had those products second-hand was 18, 22 and 10 percent respectively. These results are consistent with data and research into the second-hand market for mobile phones. In 2016 second-hand smartphones accounted for 7 percent of the global sales market and was expected to see increases in sales 4 to 5 times quicker than new phones Deliotte (2016). In addition, it has been well-documented that mobile phones are passed on to friends and family members outside of global sales, adding to the total number of second-hand devices in use. Wieser and Tröger (2016) summarises that 13 to 28 percent of all smartphones are passed onto family members or charity at their replacement point. In comparison, overall purchase rates of other second-hand small household electronics has been shown to be much lower. Overall the Pérez-Belis et al. (2017) study found only 0.75 percent of participants had bought second-hand small electronics for the home at any time where the electronics in the study included items such as vacuums, blenders, toasters and kettles. Therefore, recognising that consumer attitudes in this study showed equal interest in purchasing new robotic systems to second-hand robots with guarantees, it may be possible for the robotics industry to aim for the higher levels of second-hand device sales seen for internet-enabled devices such as mobile phones and game consoles than for the lower rates seen for standard electrical household goods.

The results of this survey were not able to show a direct relation between participants who had previously bought other second-hand electronics, and those more willing to buy second-hand robots. This may be due to ownership levels of robots for the home being so low still that the responses of participants were not influenced by their attitudes towards other types of second-hand electronics.

### 4.2 Concern factors for consumers when purchasing second-hand robots

In the Ylä-Mella et al. (2015) study of consumer perceptions towards second-hand mobile phones, concerns raised by participants as reasons for not purchasing second-hand mobile phones included reliability of the product (47 percent of respondents), short life-cycle (32 percent), availability of existing budget models (32 percent) and lack of warranty (16 percent). In comparison, this study yielded much higher concern rates, with concerns rates as high as 90 percent relating to the cost of purchasing new robots. Of the factors assessed by the Likert Scale responses in the survey, the lowest concern rate was still for robots carrying out physical damage to the home but this was still at 56 percent for new systems. Comparing ownership rates for consumer electronics within this survey only 14 percent of respondents owned a robot for the home, while 100 percent of respondents had a mobile phone. This disparity in ownership, and the relative newness of the robotics market will likely have affected concern levels for the robotic systems compared to other electronic devices. It should be noted though that even amongst robot owners in this survey, concern levels remained high, with their concern rate being 6, 8 and 10 percent higher than for those without prior robot ownership for factors of security of personal data (new), the environmental impact of manufacture (new) and cost of maintenance (2nd-hand without guarantee), highlighting the current realities of robot ownership.

Participants of the survey indicated the same levels of concern toward cost for both new and second-hand systems. This suggests that consumers may need to recognise significant cost advantages in second-hand robots in order to purchase them over new. This is reflected in the findings of Hazen et al. (2017) which highlights the need for push factors to influence the uptake by consumers in purchasing of other manufactured technology goods.

In addition to push factors affecting consumer choice in the purchase condition of electronics, pull factors such as the individual perception of environmental concerns can influence the uptake of second-hand goods Hazen et al. (2017). In this survey, 97 percent of the respondents agreed there was a climate emergency. This resulted in a too-small sample size for those that do not believe there is a climate emergency in order to compare the two demographics. However, across the survey participants, levels of concern decreased when participants considered the environmental impact involved in the manufacture of new to second-hand and second-hand without guarantee condition robots. Where consumers show a greater understanding of climate change and its impact, consumers are more likely to partake in a pro-climate consumer society Łukasz et al. (2022). The results of this survey suggest highlighting the reduction in manufacturing impact through the reuse of robotic systems should result in positive consumer behaviour towards these second-hand products. It should be noted though that in comparison, concerns about the environmental impact of disposal increased across the purchase conditions. The researchers were not able to conclude from the data why this environmental concern had an opposite result to the manufacturing concern factor. Further investigation would be needed to understand this result.

Further to the responses from the Likert Scale concern factors, the qualitative results raised additional concerns by participants around the performance of new and second-hand systems, maintainability of secondhand systems, and methods of insurance and liability of systems without a guarantee.

The topics raised as additional areas of concern in the qualitative analysis revealed that some topics were universal to robots in the home, regardless of the purchasing condition. This included the performance of the robots, and more fundamentally, trust in any robotic system coming into the home. Much as Miglani and Hensman (2016) highlights the ethical and technical considerations for software used in robots in the home, participants of this survey raised concerns regarding data security. The number of such concerns increased for second-hand systems and included issues around software obsolescence, the introduction of viruses and access to prior owners’ data. The theme of obsolescence was also raised in relation to the physical system, and the effect that buying a second-hand system might have on access to upgrades and the associated costs of upkeep. These concerns were in line with findings from research into other types of second-hand electronics. The Pérez-Belis et al. (2017) study found reasons for not purchasing second-hand electronics included cleanliness and hygiene concerns, minimal cost savings for second-hand systems over new, lack of knowledge of where to purchase second-hand devices, lack of repair guarantees and perceptions of low durability for second-hand systems. With many of these themes also appearing in this research, findings from other consumer electronic studies can be used to influence the robotics industry too.

While this study has placed emphasis on the role of the robotics industry in creating and maintaining a circular economy for products, inaction by consumers must still be considered. Pérez-Belis et al. (2017) notes that while product ecodesign is central to a circular economy and requires manufacturers to design more ‘durable, easier to repair, reuse or recycle products’, attitudes of consumers towards electronics at the end of their primary life must also be tackled. General consumers will either dispose of the e-waste in the bin instead of through dedicated WEEE recycling bins or schemes due to the relatively small size of many EEE products, or they will store the e-waste at home Pérez-Belis et al. (2017). This tendency for incorrect management of electronic waste at the end of its life was highlighted in the introduction to this paper (Section 1).

The qualitative analysis highlighted a number of participants who registered no interest in purchasing any robotic system for the home, regardless of buying condition. It is inefficient to work towards creating solutions for those unlikely to ever purchase a robot, let alone a second-hand robot. Fiorini et al. (2022) describes how participant curiosity in the technology supports greater uptake of participants; addressing this in future studies, for this topic may provide a greater and more instructive yield in results.

### 4.3 Evaluation of the surveying process

Reviewing the outcome of the participant demographics it was noted that participants with children in the home, those aged 66 and over, and those who do not believe there is a climate emergency were underrepresented in this survey in comparison to the United Kingdom population. This is a reflection that this survey partially relied on convenience sampling methods Bryman (2016)–where participants were those most available to the research team, in this case, through the publication of the request for participants via social media channels. This will likely have resulted in a more homogeneous demographic than a quota sampling system that would have produced Bryman (2016). Some effort was taken to widen the scope of participants through the use of the online Surveycircle platform, however, this platform is generally used by students and researchers, again resulting in some homogeneous traits and self-selection for participation based on interest in the topic. Results presented in Section 3 cannot, therefore, be generalised to be representative of the United Kingdom population, but do form indicating factors which was the requirement of the research process.

There were higher response levels for comments for the new robot purchase condition than second-hand conditions, despite concern levels being higher in the Likert scales for second-hand. This suggests that either the Likert Scales better-encapsulated concern factors for participants or participants spent time considering responses for this category and may not have wanted to repeat themselves for the other conditions. Only in one example did the respondent choose to copy and paste their response from one robot condition to another. The survey design for the free-text component section of the data collection could have therefore been improved by providing participants with the option to indicate the responses additionally applied to other conditions of purchase. Additionally, with limited numbers of free-text responses, motivations for purchasing habits were not explored in great detail in this paper. Future surveys could utilise follow-up interviews or additional questioning to improve on the use of free-text comments in this survey design.

While Likert Scales were used in the survey due to their ease of use and expected increased response rates, issues with using these types of scales include response acquiescence and social desirability. Acquiescence results in participants selecting results which they think are the correct answer, while social desirability causes users to select responses that make them look better for greater social acceptance Jupp (2006). Social desirability bias is often higher in surveys with an interviewer present which this research limited by utilising an online platform for data collection, however, topics around sustainability and the environment have known ethical and moral sensitivities Roxas and Lindsay (2012) which will likely have influenced participants responses.

Lastly, due to the small number of participants who submitted survey data outside of the United Kingdom, only responses from those in the United Kingdom were carried forward. Bernotat and Eyssel (2018) highlighted the effect of different cultures (Japanese and German) on the perceptions of robots in the home and attitudes while Nitto et al. (2017) studies demonstrated the differences in projected purchasing habits for consumers in the US, Japan and Germany. Future studies should therefore compare attitudes to second-hand robots for participants outside of the United Kingdom.

## 5 Conclusion

Taking survey data from 72 United Kingdom participants, around a quarter indicated a positive interest in purchasing a new robot for the home. When presented with second-hand robots with guarantees, this figure did not change. However, when the option of second-hand robots without a guarantee was introduced this was reduced to 10 percent. This highlights the need for recognised certification methods or manufacturer warranties in order for robots to be successfully sold in the second-hand market.

Young people aged 18–25 indicated a significantly higher interest in robotic systems than any other age group. Whether this is a factor of the participant’s age at the time of the survey or the generation of which they are part is unclear from this single snapshot survey. However, paired with higher interest levels in the robots presented to those who have prior experience in ownership of robotic systems, this suggests an affinity for, and greater experience with, smarter devices in the home will form key drivers for individuals being willing to purchase a consumer robot.

Current trends comparing second-hand purchases of household electronics to second-hand purchases of mobile phones suggest that the second-hand robotics market will be more similar to that of internet-enabled devices such as smartphones, than for household electronic devices–even where the robot will be used as a device for the home. By studying the growth of the second-hand mobile phone market and the challenges it has faced, the consumer robotics industry will be able to preempt the requirements that will likely be faced in trying to support the circular economy and tackle those at a time when it is cheaper in the technology maturity process to do so.

However, it should be noted that the experience of prior ownership of other types of second-hand electronic devices (such as phones, laptops and game consoles) did not increase the participant’s level of interest in second-hand robots. It is therefore possible that robotic technology for the home is too new or unknown for individuals to be able to make comparable decisions between purchasing the experience for other second-hand electronics to the projected experience of owning a robot. Instead, participants felt the initial purchase cost of any system was the greatest concern for new and second-hand robots, while the cost to maintain was the greatest concern for second-hand robots without guarantees. Additionally, attitudes to second-hand robots generally highlighted concerns for maintainability, verification and certification, technology obsolescence and liability in the event of damage to a person or home. In order to make the second-hand robot market attractive for consumers, these issues would need to be addressed and resolved, alongside a system to provide guarantees for second-hand systems. Even with these concerns though, individuals still indicated they were willing to purchase second-hand systems.

Overall this survey is encouraging for the wider implementation of the circular economy and demonstrates that there is a market for second-hand robotic systems for the home. To enable this, processes must be in place to retain consumer robots at the end of their primary use, in order to bring them into the second-hand market. As demonstrated with other electronic devices, this may require incentivisation (such as buy-back schemes), and accessible methods for consumers to return used robotic systems. Manufacturers, retailers and public systems not supporting this process will likely result in the discarding of old robots as e-waste, adding considerable levels of waste already produced annually.

## Data Availability

The datasets presented in this study can be found in online repositories. The names of the repository/repositories and accession number(s) can be found below: Due to the sensitivity of the data involved, these data are published as a restricted dataset at the University of Bristol Research Data Repository data.bris, at https://doi.org/10.5523/bris.3r4lj7jmbvekq27kljrn3ndp6u (Hauser and McGloin, 2024). The metadata record published openly by the repository at this location clearly states how data can be accessed by *bona fide* researchers. Requests for access will be considered by the University of Bristol Research Data Service, who will assess the motives of potential data re-users before deciding to grant access to the data. No authentic request for access will be refused and re-users will not be charged for any part of this process.
