# Correction: Understanding consumer attitudes towards second-hand robots for the home

**DOI:** 10.3389/frobt.2025.1694690

**Published:** 2025-09-18

**Authors:** Helen McGloin, Matthew Studley, Richard Mawle, Alan Frank Thomas Winfield

**Affiliations:** 1 FARSCOPE Centre for Doctoral Training, University of Bristol and University of West England, Bristol, United Kingdom; 2 School of Engineering, College of Arts, Technology and Environment, University of West England, Bristol, United Kingdom; 3 School of Architecture and Environment, College of Arts, Technology and Environment, University of West England, Bristol, United Kingdom

**Keywords:** robotics, sustainability, circular-economy, second-hand, e-waste, repurpose, reuse, recycle

An incorrect number was provided for the EPSRC Centre for Doctoral Training in Future Autonomous, and Robotic Systems. The correct number is **EP/S021795/1**.

The original article has been updated.

