# Supplementary material for: Understanding consumer attitudes towards second-hand robots for the home
Source: Front Robot AI. 2024 Jul 10;11:1324519. doi: 10.3389/frobt.2024.1324519 (PMC11266729; doi:10.3389/frobt.2024.1324519)
Supplement: Supplementary file 1 [file DataSheet1.pdf]

Supplementary Material

1 SUPPLEMENTARY DATA

Electronic device	New	Second-hand with guarantee	Second-hand no guarantee	N/A [a]
Mobile Phone	77.8%	12.5%	9.7%	0%
Laptop,or computer or tablet	80.6%	13.9%	4.2%	1.4%
TV or games console	65.3%	5.6%	12.5%	16.7%
Thermostats	66.7%	0%	6.9%	26.4%
Smart assistance device [b]	40.3%	0%	0%	59.7%
Internet-enabled security device	26.4%	0%	2.8%	70.8%

Notes: [a] Indicates they do not own this item, [b] eg. Amazon Echo or Google home

Table S1. Percentage of participants who bought a given electronic device based on its condition at sale

Robot Type	Purchase New	Second-hand with guarantee	Second-hand no guarantee
Entertainment	10%	14%	7%
Health & Fitness	14%	18%	8%
Household chores	64%	61%	21%
Personal Assistant	24%	22%	10%
Pet	14%	15%	3%
Security	38%	31%	10%

Table S2. Across all robot types presented, the mean percentage of participants indicating they would purchase a robot, of a given type, based on its sale condition

Robot Type	Purchase New	Second-hand with guarantee	Second-hand no guarantee
Entertainment	7%	17%	13%
Health & Fitness	31%	29%	18%
Household chores	15%	15%	25%
Personal Assistant	24%	19%	15%
Pet	11%	4%	10%
Security	24%	32%	13%

Table S3. The percentage of participants indicating they were Unsure if they would purchase a robot, of a given type, based on its sale condition

Lifestyle or demographic factor	No. Participants	Purchase New	Second-hand with guarantee	Second-hand no guarantee
All results	72	27%	27%	10%
Male	28	23%	25%	8%
Female	43	30%	28%	11%
With children	21	21%	24%	6%
Without children	51	29%	27%	11%
Aged 18-25	9	52%	41%	13%
Aged 26-35	21	29%	28%	12%
Aged 36-45	15	23%	27%	13%
Aged 46-55	8	27%	29%	0%
Aged 56-65	13	18%	18%	3%
Aged 66+	6	11%	19%	17%
Prior ownership[a]	10	32%	38%	13%
No Prior ownership[a]	61	26%	24%	9%
0 devices [b]	11	32%	32%	8%
1-3 devices [b]	34	24%	21%	9%
4-6 devices [b]	15	20%	21%	16%
7+ devices [b]	3	40%	46%	6%

[a] Prior ownership includes vacuum or mower type robotic systems. [b] Numbers of internet-enabled, connected devices already in the home

Table S4. Across all robot types presented, the mean percentage of participants of a given demographic or lifestyle factor stating they would purchase a robot, based on its sale condition

Lifestyle or demographic factor	No. Participants	Purchase New	Second-hand with guarantee	Second-hand no guarantee
All results	72	20%	20%	16%
Male	28	24%	21%	17%
Female	43	16%	18%	15%
With children	21	25%	28%	20%
Without children	51	18%	17%	14%
Aged 18-25	9	11%	20%	22%
Aged 26-35	21	16%	17%	17%
Aged 36-45	15	32%	36%	17%
Aged 46-55	8	21%	19%	17%
Aged 56-65	13	21%	13%	10%
Aged 66+	6	17%	8%	6%
Prior ownership[a]	10	30%	23%	27%
No Prior ownership[a]	61	18%	20%	14%
0 devices [b]	11	32%	32%	17%
1-3 devices [b]	34	20%	18%	10%
4-6 devices [b]	15	23%	32%	14%
7+ devices [b]	3	22%	17%	31%

[a] Prior ownership includes vacuum or mower type robotic systems. [b] Numbers of internet-enabled, connected devices already in the home

Table S5. Across all robot types presented, the mean percentage of participants of a given demographic or lifestyle factor stating they were Unsure if they would purchase a robot, based on its sale condition

Concern criteria	Robot purchase condition	Very Concerned (%)	Slightly Concerned (%)	Neutral (%)	Slightly Unconcerned (%)	Unconcerned (%)
Cost to Purchase	New	72	18	6	1	3
	2nd-hand with [a]	42	46	8	3	1
	2nd-hand without [b]	42	35	17	4	3
Cost to Maintain	New	47	36	13	3	1
	2nd-hand with	49	40	8	3	0
	2nd-hand without	60	32	7	0	1
Physical safety of people	New	29	26	19	19	6
	2nd-hand with	33	33	14	15	4
	2nd-hand without	46	24	14	11	6
Security of personal data	New	58	26	11	3	1
	2nd-hand with	61	29	3	4	3
	2nd-hand without	61	24	7	4	3
Environmental impact from manufacture	New	40	32	22	6	0
	2nd-hand with	26	36	25	13	0
	2nd-hand without	33	32	19	14	1
Environmental impact at disposal [c]	New	43	39	8	10	0
	2nd-hand with	43	35	15	6	1
	2nd-hand without	44	28	18	8	1
Physical damage to home or contents	New	33	25	26	7	8
	2nd-hand with	31	33	15	13	8
	2nd-hand without	43	28	14	11	4

[a] Second-hand with guarantee [b] Second-hand without guarantee [c] Disposal of robot at end of its useful life

Table S6. Across all robot types presented, the mean percentage of participants indicating a given concern level, based on the sale condition of a robot

Survey Question	Participant Response	No. Participants	Purchase New (%)	Second-hand with guarantee (%)	Second-hand no guarantee (%)
	All Results	72	27	27	10
Concern for e-waste levels	Concerned	56	24	26	10
	Neutral	14	67	50	0
Concern for plastic levels	Concerned	64	28	27	9
	Neutral	8	23	23	13
Condition of mobile phone purchase	New	56	29	28	10
	Refurbished with guarantee	9	13	19	11
	Second-hand no guarantee	7	31	29	2
Condition of laptop purchase	New	58	30	29	11
	Refurbished with guarantee	10	20	22	5
Condition of games console	New	47	29	28	10
	Second-hand no guarantee	9	37	35	17

Table S7. Across all robot types presented, the mean percentage of participants indicating they would purchase a robot, based on the sale condition of a robot
